# Emergency department utilization among youth in foster care: benefits of a patient-centered medical home

**DOI:** 10.3389/fped.2025.1614456

**Published:** 2025-08-29

**Authors:** Nicholas Szoko, Allison Phad, Mary E. Fournier, George M. Hoganson, Melissa Jonson-Reid

**Affiliations:** ^1^ Department of Pediatrics, Division of Adolescent Medicine, Washington University School of Medicine, St. Louis, MO, United States; ^2^ Department of Pediatrics, Division of Emergency Medicine, Washington University School of Medicine, St. Louis, MO, United States; ^3^ Washington University Brown School of Social Work, St. Louis, MO, United States

**Keywords:** foster youth, health services, emergency department (ED) utilization, patient centered medical home, trauma informed care

## Abstract

**Background:**

Foster youth face unique medical, psychological, and social challenges, which can impact patterns of emergency department (ED) utilization. The patient-centered medical home (PCMH) offers a potential model to reduce overutilization of healthcare resources. Little work has examined relationships between PCMHs and ED utilization among foster youth.

**Objectives:**

We sought to describe longitudinal trends in ED utilization among foster youth in a PCMH.

**Methods:**

This retrospective cohort study leveraged electronic health record data from foster youth seen in a PCMH from 2018 to 2024. Frequency of ED utilization was calculated before, during, and after involvement in the PCMH. We used Poisson generalized linear mixed models to examine associations between each time period (before, during, or after) and the frequency of ED visits, adjusting for age, sex, and race/ethnicity. Results are presented as incidence rate ratios (IRR) with 95% confidence intervals (95% CI).

**Results:**

Out of 857 youth, 525 (61.3%) were female. Median age was 15.5 years (IQR: 14.2–16.8). Most youth were Black or African American (*n* = 643; 75.0%). The median number of ED visits was three (IQR: 2–7) for the total observation period. Compared to before the PCMH, ED utilization was significantly lower during engagement with the PCMH [IRR: 0.62 (95% CI: 0.54–0.70)]. ED utilization increased after the last PCMH encounter [IRR: 1.12 (95% CI: 1.01–1.24)].

**Conclusions:**

Engagement in a PCMH was associated with lower ED utilization among foster youth. Findings suggest that PCMHs may decrease healthcare costs and better support foster youths’ specific health needs.

## Introduction

In 2022, over 500,000 youth were involved in foster care system nationwide (1). Youth in foster care experience unique health challenges, such as high mental health morbidity, foregone preventative care, and unmet sexual and reproductive health needs (2). These issues may be even greater for older youth (i.e., adolescents) in foster care, who face additional risks related to “aging out” of the system (3). Black youth and youth identifying as sexual and gender minorities are more likely to enter foster care (4, 5), disparities driven by disproportionate exposure to trauma, discrimination, and poverty (6–8). Thus, strategies to support youth in foster care must adopt healing-centered approaches that acknowledge these histories and provide holistic supports to buffer against negative health outcomes. Further, addressing these multi-layered health inequities requires models of care that are not only accessible, but integrative and developmentally attuned.

One potential model to better meet the health needs of this unique population is the patient-centered medical home (PCMH) (9). PCMHs, which integrate preventative and acute medical care, behavioral health, and case management services, can provide intensive medical and psychosocial supports within a unified setting (10). This strategy reduces key barriers in care access, such as transportation to and from appointments and coordinating visits across multiple providers, and the longitudinal nature of services facilitates trust-building with practitioners. In addition, PCMHs may decrease overutilization of healthcare resources, such as emergency department (ED) visits for non-emergent conditions (11), which can decrease associated healthcare costs. This is particularly relevant for youth in foster care, who, despite comprising less than 2% of Medicaid enrollees (12), incur a disproportionate burden of Medicaid costs (13).

Prior research has demonstrated that youth in foster care have higher rates of hospitalization and subspecialty care, as well as greater health service costs, compared to their peers without foster system involvement (14). This may be due, in part, to a greater number of visits for psychiatric concerns, which account for a large proportion of health service utilization in this population (15). Traumatic stress may also increase somatic symptoms (e.g., pain) due to overactivation of pro-inflammatory pathways and the glucocorticoid response (16, 17). In addition, foster youth may experience more foregone healthcare prior to entry into the system (18, 19). Factors such as placement instability and insurance discontinuity may also contribute (20–22). Data regarding ED utilization among foster youth is mixed. While past-year ED utilization may be as high as 31% among foster youth (23), some evidence suggests youth in foster care are less likely to seek care for preventable medical concerns (24). However, studies involving mental health diagnoses consistently demonstrate increased care utilization for foster youth, which reflects the complex psychosocial challenges they encounter (25–27). Although PCMHs may provide a mechanism to reduce healthcare costs in foster youth (28), studies evaluating impacts of the PCMH on ED utilization in foster youth is limited.

The goal of the present study was to evaluate trends in ED utilization among foster youth by leveraging electronic health record data from an academic-affiliated PCMH from 2018 to 2024. We specifically sought to examine whether 1) ED utilization decreased during the period adolescents received care through the PCMH and 2) ED utilization remained lower following their transition out of the PCMH. In doing so, we aimed to assess whether a PCMH specific to foster youth may reduce ED overutilization in this unique population.

## Methods

### Study design, sample, and data source

This retrospective cohort study leveraged data obtained from the electronic medical record of patients receiving care at the Creating Options and Choosing Health (COACH) clinic, a PCMH for foster youth aged 12–25 years in St. Louis City and St. Louis County. In the state of Missouri, all youth between 12 and 17 years of age who enter foster care are required to undergo a comprehensive medical evaluation within 30 days of placement, which usually occurs at the COACH program. Foster youth who are older than age 17, including those who have aged out, remain eligible for clinic services and can be referred at any time.

COACH uses an interdisciplinary, team-based approach for each visit involving a nurse, physician or advanced practice provider, and a case manager. Case managers communicate regularly with patients, foster parents, and case workers to ensure access to needed medical care (e.g., subspecialty referrals), while offering connections to psychosocial resources (e.g., education, employment, housing). In addition to primary care, COACH provides free and confidential sexual and reproductive health services to all patients, as well as embedded behavioral health supports (e.g., psychiatry and therapy), housed in the same space. COACH utilizes a youth-centered approach, in which the majority of the visit occurs individually with the youth. All clinical and educational materials are designed to be youth-friendly with age-appropriate language and simplified visual aids.

For the present study, our cohort was identified by selecting individuals who attended a new foster care visit between the ages of 12–25 years from January 1st, 2018 (when our clinic transitioned to the current electronic medical record) to December 31st, 2024. After identifying this cohort, we abstracted patient-level demographics and encounter-specific data as described below. This study was approved by the Washington University Institutional Review Board.

### Measures

Demographic data included patient age at their initial visit in the foster care clinic, sex at birth, race, and ethnicity. For encounter-specific data, we utilized all outpatient and ED encounters for the patient sample between 2018 and 2024. Encounters included visit date, visit type (i.e., foster care clinic, ED, or other outpatient), and visit diagnoses. Encounter data was linked to patient-level demographics using medical record numbers. In order to assess ED utilization before, during, and after engagement with our clinic, we first defined these time periods as follows: (1) before: time from first encounter of any type to first visit with foster care clinic; (2) during: time from first visit with foster care clinic to last visit with foster care clinic; (3) after: time from last visit with foster care clinic to last encounter of any type. Individuals without any encounter data before or after their involvement in our clinic were excluded from analysis, as it was assumed that they were likely to seek ED care through another system, and therefore utilization based on censoring would be unreliable. ED utilization was calculated by determining the count of ED visits in the period before, during, and after engagement with the foster care clinic for each patient. There were 1,373 unique diagnoses across ED visits; to allow for meaningful interpretation, visit diagnoses were manually coded into illness categories by the principal investigator (NS) using a deductive approach. Subcodes within each category are presented as a supplement (Supplementary Table S1).

### Statistical analysis

Demographic characteristics for the entire sample were summarized with medians and interquartile ranges (IQR) and frequencies and proportions for continuous and categorical variables, respectively. ED diagnosis categories were tabulated for the overall sample and for high utilizers, defined as youth with more than 15 visits (third quartile + 1.5*IQR) in the observation period (29). To assess whether ED utilization varied over the course of engagement with our clinic, we used generalized linear mixed models with Poisson distributions to examine associations between time period (before, during, or after) and the count of ED visits, with random intercepts to account for repeated measures within participants. Models included an offset (i.e., days observed for each time period) to account for differential observation times, as youth observed for longer durations would be expected to have more ED visits. Models additionally adjusted for age at initial COACH visit, sex at birth, and race/ethnicity (collapsed into a three-level categorical variable: non-Hispanic Black, non-Hispanic White, and Other). Model fit was assessed by visualizing deviance residuals. Due to the presence of several outliers in our initial model, we excluded observations (*n* = 86; 3.3%) with residual values >2. Models showed no evidence of overdispersion. Given potential changes in utilization related to COVID-19, as a sensitivity analysis, we stratified analyses based on having an initial visit before or on/after January 1st, 2020. We present incidence rate ratios (IRR) comparing ED utilization during and after engagement with COACH to ED utilization before COACH and 95% Wald confidence intervals (CI). All analyses were conducted in R version 4.4.1.

## Results

### Youth characteristics

A total of 857 youth were included in the sample (Table 1). Median age at initial COACH visit was 15.5 years (IQR: 14.2–16.8). In terms of sex at birth, 525 (61.3%) were female and 332 (38.7%) were male. Most youth were Black or African American (*n* = 643; 75.0%) or White (*n* = 170; 19.8%), with fewer reporting other racial identities. Twenty-nine youth (3.4%) were of Hispanic or Latino origin. The median number of COACH encounters was 4 (IQR: 2–12) for youth in the sample. Out of the 857 youth seen for an initial COACH visit, most (*n* = 691; 80.6%) returned for follow-up. Youth remained engaged with COACH for a median of three months (IQR: 0–18).

**Table 1:** Demographic characteristics of sample: 2018–2024.

Characteristic	Overall (*n* = 857)	Typical utilizers (*n* = 809)	High utilizers^a^ (*n* = 48)
Age at initial encounter: years	15.5 (14.2–16.8)	15.4 (14.2–16.8)	16.1 (14.5–17.5)
Sex			
Female	525 (61.3%)	488 (60.3%)	37 (77%)
Male	332 (38.7%)	321 (39.7%)	11 (23%)
Race			
American Indian or Alaska Native	1 (0.1%)	1 (0.1%)	0 (0%)
Asian	3 (0.4%)	3 (0.4%)	0 (0%)
Black or African American	643 (75.0%)	597 (73.8%)	46 (96%)
Native Hawaiian or Other Pacific Islander	2 (0.3%)	2 (0.2%)	0 (0%)
Multiracial/Other	18 (2.2%)	18 (2.2%)	0 (0%)
White	170 (19.8%)	168 (20.8%)	2 (4%)
Missing/Unknown	20 (2.3%)	20 (2.5%)	0 (0%)
Ethnicity: Hispanic or Latino	29 (3.4%)	29 (3.6%)	0 (0%)
Number of COACH visits			
Median per youth	4 (2–12)	4 (2–12)	4 (2–14)
Youth with at least one return visit	691 (80.6%)	652 (80.6%)	39 (81%)

^a^ Defined as youth with >15 emergency department visits during observation period.

### Emergency department visits

The median number of ED visits was three (IQR: 2–7) for the observation period and one (IQR: 0–2), zero (IQR: 0–1), and one (IQR: 0–3) before, during, and after COACH, respectively. Out of 3,767 total ED encounters (Table 2), the most common visit diagnoses were related to: infection (e.g., pneumonia, gastroenteritis, sexually transmitted infections) (*n* = 759; 20.1%); psychiatric illness (e.g., suicidality, aggression, psychosis) (*n* = 708; 18.8%); and injury/trauma (e.g., fractures, lacerations, motor vehicle collisions) (*n* = 615; 16.3%). Visits due to chronic medical conditions (i.e., asthma, diabetes, sickle cell disease) represented a small proportion of visits (*n* = 157; 4.2%). Out of 857 youth in the sample, 48 (5.6%) were categorized as high utilizers (Table 1). Visit reasons were similar to those observed in the overall sample, though high utilizers had a greater proportion of visits with pain-related diagnoses (e.g., abdominal pain, chest pain, vaso-occlusive crises) (Table 2).

**Table 2:** Emergency department visits by diagnosis group.

Diagnosis category	Overall (*n* = 3,767)	Typical utilizers (*n* = 2,412)	High utilizers (*n* = 1,355)
Allergic reaction	36 (1%)	30 (1.2%)	6 (0.4%)
Cardiovascular	9 (0.2%)	7 (0.3%)	2 (0.1%)
Gastrointestinal	103 (2.7%)	63 (2.6%)	40 (3%)
Genitourinary	121 (3.2%)	66 (2.7%)	55 (4.1%)
Hematologic	6 (0.2%)	3 (0.1%)	3 (0.2%)
Infection	759 (20.1%)	459 (19%)	297 (21.9%)
Injury	615 (16.3%)	461 (19.1%)	154 (11.4%)
Lab abnormality	4 (0.1%)	4 (0.2%)	40 (3%)
Metabolic^a^	82 (2.2%)	42 (1.7%)	46 (3.4%)
Neurologic	120 (3.2%)	74 (3.1%)	192 (14.2%)
Pain^b^	453 (12%)	264 (10.9%)	52 (3.8%)
Pregnancy	122 (3.2%)	70 (2.9%)	231 (17%)
Psychiatric & behavioral	708 (18.8%)	477 (19.8%)	41 (3%)
Respiratory^c^	107 (2.8%)	66 (2.7%)	14 (1%)
Skin	41 (1.1%)	27 (1.1%)	47 (3.5%)
Social	129 (3.4%)	82 (3.4%)	7 (0.5%)
Well care	57 (1.5%)	50 (2.1%)	128 (9.4%)
Left/Not seen/Unknown	295 (7.8%)	167 (6.9%)	6 (0.4%)

^a^ Included 80 visits due to diabetes (*n* = 38 for high utilizers).

^b^ Included 14 visits due to vaso-occlusive crises (*n* = 13 for high utilizers).

^c^ Included 63 visits due to asthma (*n* = 26 for high utilizers).

### Trends in emergency department utilization

Compared to before COACH, ED utilization was significantly lower during engagement with the COACH program [IRR: 0.62 (95% CI: 0.54–0.70)] (Table 3). ED utilization increased after the last encounter with COACH [IRR: 1.12 (95% CI: 1.01–1.24)]. In adjusted models, higher age at initial encounter [IRR: 1.03 (95% CI: 1.00–1.06)], female sex [IRR: 1.23 (95% CI: 1.05–1.42)], and non-Hispanic Black identity [IRR: 1.30 (95% CI: 1.05–1.61), reference: non-Hispanic White] were associated with higher ED utilization overall. Findings were consistent before and after the start of the COVID-19 pandemic with one exception—for youth with an initial visit before the start of the pandemic, ED utilization did not significantly increase after engagement with COACH [IRR: 0.92 (95% CI: 0.78–1.08)].

**Table 3:** Summary of ED utilization before, during, and after involvement with COACH.

Measure	Before	During	After
Number of ED visits: Median (IQR)	1 (0–2)	0 (0–1)	1 (0–3)
Number of ED visits: Mean (SD)	3 (7)	2 (5)	5 (14)
Observation time (months): Median (IQR)	3 (0–22)	3 (0–18)	2 (0–24)
Incidence rate ratio (95% CI)	Ref	0.62 (0.54–0.70)***	1.12 (1.01–1.24)*

^a^ Results from generalized linear mixed models adjusted for age at initial visit, race/ethnicity, and sex at birth with random intercept for patient.

*** *p* < 0.001; *p = 0.05.

## Discussion

In this retrospective study of foster youth engaged in a PCMH, we found significant reductions in ED utilization during involvement with the program. This aligns with prior work that has shown the benefits of PCMH for individuals with medical complexity and chronic disease. By offering integrated medical and behavioral health care with intensive case management support, PCMHs may serve as a critical touchpoint for preventing foregone care or disease progression in a particularly vulnerable subgroup. As such, resultant costs associated with higher levels of care may be avoided. Consistent with prior literature, we noted a high proportion of ED visits related to behavioral health concerns, and we found that some youth were high utilizers across all time periods of the study. However, we also observed that for the entire sample, rates of ED utilization *increased* after leaving the PCMH, even relative to rates prior to involvement in the program.

This pattern may be related to the unique challenges associated with entry into young adulthood. Youth transitioning out of foster care are at high risk of housing instability, unemployment, justice system involvement, and poverty (30, 31). All of these factors may contribute to greater health needs and create structural barriers to medical follow-up. Indeed, youth aging out of foster care have higher rates of mental health challenges and substance use (32, 33), yet are less likely to engage in outpatient mental health treatment (34). Given this especially vulnerable period, many states have implemented policies to allow extension of foster care beyond age 18 (35), and numerous agencies have developed multilevel interventions aimed at supporting this subgroup (36). Our findings reaffirm the critical importance of caring for youth beyond their involvement in the system. Although our PCMH is open to current or former foster youth, there may be opportunities to strengthen supports for patients entering young adulthood through dedicated transition planning, continued case management support, or integration and/or co-location of adult providers. In addition, it is essential to bolster known protective factors among foster youth transitioning out of the system, such as safe and caring relationships, nonfamilial mentoring, and setting goals for the future (37–39).

Similar to other studies in this population, we demonstrated that most ED visits were related to behavioral health concerns, such as suicidality and aggression. Beyond having a higher burden of mental health morbidity (40), youth in foster care may also be impacted by the overall shortage of pediatric mental health services globally (41). Prior work has demonstrated that increased age, as well as delayed receipt of mental health treatment services, may contribute to ED utilization for this purpose (15, 42). Thus a PCMH, particularly one which includes psychiatric services and trauma-focused therapy, may be particularly well-suited to address this issue by decreasing time to entry into care. This aligns with qualitative research with foster youth and their families (43), where integrating mental health services with primary care was felt to be valuable. Furthermore, both longitudinal trust-building and provider continuity have been cited as key elements of ongoing service engagement, especially in preparing foster youth for the transition to adult mental health care (44). The PCMH offers an ideal setting for this occur when tailored to the unique developmental needs of adolescence.

Youth that were older, female, and non-Hispanic Black had higher ED utilization overall. However, among the 48 youth who were categorized as being high utilizers, we noted similarities in diagnostic categories, with the exception of pain. This may be due, in part, to known disparities in pain management according to racial identity, in which Black patients are less likely to receive analgesia (45). If pain control is inadequate, these individuals may return more frequently to seek care. Although some studies demonstrate increased ED utilization among individuals with functional pain disorders (i.e., in the setting of comorbid depression or anxiety) (46), Black women in particular are *less* likely to report these somatic symptoms than other identities (47, 48), making it essential for providers to combat structural racism and interpersonal bias when providing clinical care (49). This is of added importance for youth with foster system involvement, where experiences of discrimination may be even more common. PCMHs offer an ideal setting to incorporate healing-centered frameworks (50), which acknowledge these histories and cultivate well-being through relationship-building, hope, and empowerment.

There are several important limitations to this work which must be considered. First, as data was limited to information extracted from the electronic medical record, we were not able to account for important sources of confounding, such as placement changes and insurance discontinuity. In addition, we only examined data within one electronic medical interface, which may miss ED encounters occurring outside of a partnering health system, or care sought in other settings (e.g., urgent care). These variables may be inherently linked; youth with greater placement instability may be more likely to seek care outside of a single health system. Importantly, PCMH models have the potential to bridge care across multiple settings. This informed eligibility for COACH, which allows youth to remain in care through age 25, regardless of ongoing involvement in the foster care system.

That said, because our program focuses specifically on adolescents, our findings may not be generalizable to younger children in the foster system. Involvement in COACH was not randomized, so individuals engaged in the program may systematically differ from other youth in foster care. While the racial and ethnic makeup of our PCMH mirrors known disparities in the larger foster care system (51), the lack of reliable sexual orientation and gender identity data in our medical record prevents us from being able to assess all aspects of identity. This is a key gap in light of known systemic inequities for youth who identify as sexual or gender minority (4, 5). Applying an intersectional lens to future analyses is critical to better identify patterns of overutilization which can be addressed with tailored interventions (52, 53). We also noted a higher prevalence of females in our sample, suggesting additional structural barriers for males, such as crossover into the juvenile justice system (54).

We did not exclude non-preventable ED visits, and it is likely that some visits related to chronic medical illness were misclassified due to primary diagnostic code (e.g., “chest pain” rather than “status asthmaticus”). Greater research is needed to examine whether decreased ED utilization rates for preventable reasons are directly linked to PCMH visits. This has important implications regarding cost effectiveness analyses, as many health problems in this population may persist into adulthood. Despite these limitations, we were able to assess care utilization in a relatively large sample without reliance on self-report, which overcomes some of the gaps in prior literature. In addition, we observed youth over a relatively long follow-up period, enabling us to track utilization patterns after leaving the PCMH.

Together, our findings highlight the potential for PCMH to better support the unique medical, psychological, and social needs of youth in foster care. Rather than viewing the PCMH as a cost-saving strategy alone, our findings suggest it serves as a stabilizing institution that merits continued investment, particularly during transitions to adulthood. Future work should incorporate multilevel cost effectiveness analyses to understand broader implications for health services spending. This may involve identifying the “core components” of a successful PCMH intervention. In addition, describing care utilization across multiple settings (e.g., inpatient, residential, other outpatient) may provide a more comprehensive picture regarding use patterns in this population. Overall, our findings underscore the importance of minimizing barriers to care access, building longitudinal relationships, and creating trustworthy clinical environments for youth in foster care to better support well-being.

## Data Availability

The original contributions presented in the study are included in the article/Supplementary Material, further inquiries can be directed to the corresponding author.

## Supplementary material

Supplementary Table S1 Detailed breakdown of diagnostic categories across ED visits (*n* = 3,767).

## References

[1] Administration for Children and Families. The AFCARS Report. Washington, DC: U.S. Department of Health and Human Services (2024). Available online at: https://acf.gov/cb/report/afcars-report-30

[2] Szilagyi MA Rosen DS Rubin D Zlotnik S. Health care issues for children and adolescents in foster care and kinship care. Pediatrics. (2015) 136(4):e1142–66. 10.1542/peds.2015-2656 26416934

[3] Jaudes PK Szilagyi MA Fierson W Harmon DA High PE Jones VF Health care of youth aging out of foster care. Pediatrics. (2012) 130(6):1170–3. 10.1542/peds.2012-2603 23184106

[4] Baams L Wilson BDM Russell ST. LGBTQ youth in unstable housing and foster care. Pediatrics. (2019) 143(3):e20174211. 10.1542/peds.2017-4211 30745432 PMC6398424

[5] Fish JN Baams L Wojciak AS Russell ST. Are sexual minority youth overrepresented in foster care, child welfare, and out-of-home placement? Findings from nationally representative data. Child Abuse Neglect. (2019) 89:203–11. 10.1016/j.chiabu.2019.01.005 30708335 PMC7306404

[6] Salazar AM Keller TE Gowen LK Courtney ME. Trauma exposure and PTSD among older adolescents in foster care. Soc Psychiatry Psychiatr Epidemiol. (2013) 48(4):545–51. 10.1007/s00127-012-0563-0 22898825 PMC4114143

[7] Rebbe R Nurius PS Ahrens KR Courtney ME. Adverse childhood experiences among youth aging out of foster care: a latent class analysis. Child Youth Serv Rev. (2017) 74:108–16. 10.1016/j.childyouth.2017.02.004 28458409 PMC5404688

[8] Rebbe R Nurius PS Courtney ME Ahrens KR. Adverse childhood experiences and young adult health outcomes among youth aging out of foster care. Acad Pediatr. (2018) 18(5):502–9. 10.1016/j.acap.2018.04.011 29709622 PMC6035089

[9] Jackson GL Powers BJ Chatterjee R Prvu Bettger J Kemper AR Hasselblad V The patient-centered medical home. Ann Intern Med. (2013) 158(3):169–78. 10.7326/0003-4819-158-3-201302050-00579 24779044

[10] Stange KC Nutting PA Miller WL Jaén CR Crabtree BF Flocke SA Defining and measuring the patient-centered medical home. J Gen Intern Med. (2010) 25(6):601–12. 10.1007/s11606-010-1291-3 20467909 PMC2869425

[11] David G Gunnarsson C Saynisch PA Chawla R Nigam S. Do patient-centered medical homes reduce emergency department visits? Health Serv Res. (2015) 50(2):418–39. 10.1111/1475-6773.12218 25112834 PMC4369216

[12] Medicaid.gov. Improving Timely Health Care for Children and Youth in Foster Care. Baltimore, MD: Centers for Medicare & Medicaid Services (2024). Available online at: https://www.medicaid.gov/medicaid/quality-of-care/quality-improvement-initiatives/foster-care-learning-collaborative/index.html#one

[13] Rosenbach M Lewis K Quinn B, ASPE. Health Conditions, Utilization, and Expenditures of Children in Foster Care. Washington, DC: U.S. Department of Health and Human Services (1999). Available online at: http://aspe.hhs.gov/reports/health-conditions-utilization-expenditures-children-foster-care (Accessed March 17, 2025)

[14] Bennett CE Wood JN Scribano PV. Health care utilization for children in foster care. Acad Pediatr. (2020) 20(3):341–7. 10.1016/j.acap.2019.10.004 31622784

[15] Vish NL Budig K Stolfi A Elliston R Thackeray JD. Predictors of mental health emergency department visits and psychiatric hospitalizations in children in foster care. Child Youth Serv Rev. (2024) 158:107485. 10.1016/j.childyouth.2024.107485

[16] Herzog JI Schmahl C. Adverse childhood experiences and the consequences on neurobiological, psychosocial, and somatic conditions across the lifespan. Front Psychiatry. (2018) 9:420. 10.3389/fpsyt.2018.00420 30233435 PMC6131660

[17] Kugler BB Bloom M Kaercher LB Truax TV Storch EA. Somatic symptoms in traumatized children and adolescents. Child Psychiatry Human Dev. (2012) 43(5):661–73. 10.1007/s10578-012-0289-y 22395849

[18] Steele JS Buchi KF. Medical and mental health of children entering the Utah foster care system. Pediatrics. (2008) 122(3):e703–9. 10.1542/peds.2008-0360 18762506

[19] Deutsch SA Fortin K. Physical health problems and barriers to optimal health care among children in foster care. Curr Probl Pediatr Adolesc Health Care. (2015) 45(10):286–91. 10.1016/j.cppeds.2015.08.002 26364980

[20] Beal SJ Ammerman RT Mara CA Nause K Greiner MV. Patterns of healthcare utilization with placement changes for youth in foster care. Child Abuse Negl. (2022) 128:105592. 10.1016/j.chiabu.2022.105592 35334304 PMC11697974

[21] Day A Curtis A Paul R Allotey PA Crosby S. Timely health service utilization of older foster youth by insurance type. J Adolesc Health. (2016) 58(1):17–23. 10.1016/j.jadohealth.2015.09.019 26707226

[22] Fawley-King K Snowden LR. Relationship between placement change during foster care and utilization of emergency mental health services. Child Youth Serv Rev. (2012) 34(2):348–53. 10.1016/j.childyouth.2011.11.002

[23] Jee SH Antonucci TC Aida M Szilagyi MA Szilagyi PG. Emergency department utilization by children in foster care. Ambul Pediatr. (2005) 5(2):102–6. 10.1367/A04-068R.1 15780011

[24] Malthaner LQ McLeigh JD Knell G Jetelina KK Atem F Messiah SE. Preventable emergency department utilization among patients with foster care history compared to patients without foster care history. Child Maltreat. (2024):10775595241300971. 10.1177/10775595241300971 39585789 PMC12495112

[25] MacDonald K Laporte L Desrosiers L Iyer SN. Emergency department use for mental health problems by youth in child welfare services. J Can Acad Child Adolesc Psychiatry. (2022) 31(4):202–13. 36425014 PMC9661906

[26] Shin SH. Need for and actual use of mental health service by adolescents in the child welfare system. Child Youth Serv Rev. (2005) 27(10):1071–83. 10.1016/j.childyouth.2004.12.027

[27] Harman JS Childs GE Kelleher KJ. Mental health care utilization and expenditures by children in foster care. Arch Pediatr Adolesc Med. (2000) 154(11):1114. 10.1001/archpedi.154.11.1114 11074852

[28] Gannon BS Gregg A Wang H Marshall ME Yerby LG Jenkins C A medical home for children in foster care reduces expenditures. Childrens Health Care. (2022) 51(3):285–99. 10.1080/02739615.2022.2039146

[29] Larson MG. Descriptive statistics and graphical displays. Circulation. (2006) 114(1):76–81. 10.1161/CIRCULATIONAHA.105.584474 16818830

[30] Gypen L Vanderfaeillie J De Maeyer S Belenger L Van Holen F. Outcomes of children who grew up in foster care: systematic-review. Child Youth Serv Rev. (2017) 76:74–83. 10.1016/j.childyouth.2017.02.035

[31] Berzin SC Rhodes AM Curtis MA. Housing experiences of former foster youth: how do they fare in comparison to other youth? Child Youth Serv Rev. (2011) 33(11):2119–26. 10.1016/j.childyouth.2011.06.018

[32] Lopez P Allen PJ. Addressing the health needs of adolescents transitioning out of foster care. Pediatr Nurs. (2006) 33(4):345–55. 17907736

[33] Havlicek JR Garcia AR Smith DC. Mental health and substance use disorders among foster youth transitioning to adulthood: past research and future directions. Child Youth Serv Rev. (2013) 35(1):194–203. 10.1016/j.childyouth.2012.10.003 23766549 PMC3677527

[34] McMillen JC Raghavan R. Pediatric to adult mental health service use of young people leaving the foster care system. J Adolesc Health. (2009) 44(1):7–13. 10.1016/j.jadohealth.2008.04.015 19101453 PMC2633876

[35] Child Welfare Information Gateway. Extension of Foster Care Beyond Age 18. Washington, DC: U.S. Department of Health and Human Services, Administration for Children and Families, Children's Bureau (2022). Available online at: https://www.childwelfare.gov/resources/extension-foster-carebeyond-age-18/

[36] Greeson JKP Garcia AR Tan F Chacon A Ortiz AJ. Interventions for youth aging out of foster care: a state of the science review. Child Youth Serv Rev. (2020) 113:105005. 10.1016/j.childyouth.2020.105005

[37] Courtney ME Piliavin I Grogan-Kaylor A Nesmith A. Foster youth transitions to adulthood: a longitudinal view of youth leaving care. Child Welfare. (2001) 80(6):685–717. https://www.jstor.org/stable/45400302 11817658

[38] Geenen S Powers LE. “Tomorrow is another problem”: the experiences of youth in foster care during their transition into adulthood. Child Youth Serv Rev. (2007) 29(8):1085–101. 10.1016/j.childyouth.2007.04.008

[39] Nuñez M Beal SJ Jacquez F. Resilience factors in youth transitioning out of foster care: a systematic review. Psychological trauma: theory, research. Pract Policy. (2022) 14(S1):S72–81. 10.1037/tra0001096 PMC9070687 34582226

[40] Oswald SH Heil K Goldbeck L. History of maltreatment and mental health problems in foster children: a review of the literature. J Pediatr Psychol. (2010) 35(5):462–72. 10.1093/jpepsy/jsp114 20007747

[41] Benton TD Boyd RC Njoroge WFM. Addressing the global crisis of child and adolescent mental health. JAMA Pediatr. (2021) 175(11):1108–10. 10.1001/jamapediatrics.2021.2479 34369961

[42] Gibbs DJ Konstanzer J Hassmiller Lich K Lanier P Ansong D Chapman MV Mental health treatment delays for youth in foster care: understanding system decisions and dynamics. Adm Policy Ment Health. (2025) 52:542–60. 10.1007/s10488-025-01432-3 39945935

[43] Jee SH Conn AM Toth S Szilagyi MA Chin NP. Mental health treatment experiences and expectations in foster care: a qualitative investigation. J Public Child Welf. (2014) 8(5):539–59. 10.1080/15548732.2014.931831

[44] Broad KL Sandhu VK Sunderji N Charach A. Youth experiences of transition from child mental health services to adult mental health services: a qualitative thematic synthesis. BMC Psychiatry. (2017) 17(1):380. 10.1186/s12888-017-1538-1 29183289 PMC5706294

[45] Lee P Le Saux M Siegel R Goyal M Chen C Ma Y Racial and ethnic disparities in the management of acute pain in US emergency departments: meta-analysis and systematic review. Am J Emerg Med. (2019) 37(9):1770–7. 10.1016/j.ajem.2019.06.014 31186154

[46] Garde V Thornton K Pardon M Gangathimmaiah V Mallett AJ Greenslade J Functional somatic symptoms in emergency department frequent presenters. BMC Emerg Med. (2024) 24(1):122. 10.1186/s12873-024-01030-w 39020282 PMC11256397

[47] Sauceda JA Patel AR Santiago-Rodriguez EI Garcia D Lechuga J. Testing for differences in the reporting of somatic symptoms of depression in racial/ethnic minorities. Health Educ Behav. (2021) 48(3):260–4. 10.1177/10901981211011925 34080483

[48] Lara-Cinisomo S Akinbode TD Wood J. A systematic review of somatic symptoms in women with depression or depressive symptoms: do race or ethnicity matter? J Womens Health. (2020) 29(10):1273–82. 10.1089/jwh.2019.7975 32397866

[49] Agarwal AK Gonzales RE Sagan C Nijim S Asch DA Merchant RM Perspectives of black patients on racism within emergency care. JAMA Health Forum. (2024) 5(3):e240046. 10.1001/jamahealthforum.2024.0046 38457129 PMC10924244

[50] Ginwright S. The future of healing: shifting from trauma informed care to healing centered engagement. Occasion Paper. (2018) 25:25–32.

[51] The Annie E, Casey Foundation, Kids Count Data Center. Children in Foster Care by Race and Hispanic Origin in the United States. Baltimore, MD: Annie E. Casey Foundation (2022). Retrieved from www.aecf.org

[52] Crenshaw K. Mapping the margins: intersectionality, identity politics, and violence against women of color. Stanford Law Rev. (1991) 43(6):1241. 10.2307/1229039

[53] Rosenthal L. Incorporating intersectionality into psychology: an opportunity to promote social justice and equity. Am Psychol. (2016) 71(6):474. 10.1037/a0040323 27571527

[54] Jonson-Reid M Dunnigan A Ryan J. Foster care and juvenile justice systems: crossover and integration of services. Handbook of Foster Youth. New York, NY: Routledge (2018). p. 456–72.

