# Supplementary material for: Emergency department utilization among youth in foster care: benefits of a patient-centered medical home
Source: Front Pediatr. 2025 Aug 29;13:1614456. doi: 10.3389/fped.2025.1614456 (PMC12425793; doi:10.3389/fped.2025.1614456)
Supplement: Supplementary Table S1 — Detailed breakdown of diagnostic categories across ED visits (n = 3,767). [file Table1.pdf]

eTable 1. Detailed breakdown of diagnostic categories across ED visits (n=3,767)

Diagnosis Category	Example Diagnoses	Count (%)
**Allergic Reaction**	Allergic conjunctivitis, allergic rhinitis, anaphylaxis	36 (1%)
**Cardiovascular**	Cardiac arrest, palpitations, pericarditis	9 (0.2%)
**Gastrointestinal**	Constipation, diarrhea, nausea	103 (2.7%)
**Genitourinary**	Amenorrhea, dysuria, hematuria	121 (3.2%)
**Hematologic**	Anemia	6 (0.2%)
**Infection**		759 (20.1%)
Gastrointestinal	Appendicitis, gastroenteritis, infectious diarrhea	22 (0.6%)
Genitourinary	Acute pelvic inflammatory disease, bacterial vaginosis, exposure to sexually transmitted disease	337 (8.9%)
Other	Fever, influenza, viral illness	85 (2.3%)
Respiratory	Bronchitis, laryngitis, pneumonia	240 (6.4%)
Skin	Cellulitis, folliculitis, paronychia	67 (1.8%)
**Injury**	Abrasion, contusion, fracture, laceration, sprain, strain	615 (16.3%)
**Lab Abnormality**	Elevated CK, elevated INR, transaminitis	4 (0.1%)
**Metabolic**		82 (2.2%)
Metabolic	Hypothyroidism, ketosis	2 (0.1%)
Diabetes	Diabetic ketoacidosis, hyperglycemia, type 1 diabetes mellitus	80 (2.1%)
**Neurologic**	Dizziness, weakness, seizure	120 (3.2%)
**Pain**		453 (12%)
Cardiovascular	Chest pain	44 (1.2%)
Dental	Caries, tooth pain	18 (0.5%)
Gastrointestinal	Abdominal cramping, abdominal pain	154 (4.1%)
Genitourinary	Groin pain, vaginal pain	11 (0.3%)
Musculoskeletal	Hand pain, knee pain, foot pain	137 (3.6%)
Neurologic	Headache, migraine	61 (1.6%)
Other	Post-op pain	1 (0%)
Respiratory	Ear pain, eustachian tube dysfunction, otalgia	4 (0.1%)
Skin	Itching, nipple tenderness, scalp pain	9 (0.2%)
Sickle Cell	Sickle cell pain crisis	14 (0.4%)
**Pregnancy**	Bleeding in pregnancy, incomplete abortion, miscarriage, positive pregnancy test	122 (3.2%)
**Psychiatric & Behavioral**	Aggression, depression, homicidal ideation, schizophrenia, suicidal ideation	708 (18.8%)
**Respiratory**		107 (2.8%)
Respiratory	Cough, dyspnea, tachypnea	44 (1.2%)
Asthma	Asthma exacerbation, status asthmaticus	63 (1.7%)
**Skin**	Acne vulgaris, hydradenitis, rash	41 (1.1%)
**Social**		129 (3.4%)
Abuse/Neglect	Child abuse, child neglect, sexual abuse	46 (1.2%)
Social	Family conflict, housing instability, poor social situation	83 (2.2%)
Well Care	Encounter for medical screening examination, encounter for well adolescent visit, normal exam	57 (1.5%)
